# Exploring physiotherapy practice within hospital-based interprofessional chronic pain clinics in Ontario

**DOI:** 10.1080/24740527.2021.1905508

**Published:** 2021-04-29

**Authors:** Linnea Thacker, Robert M. Walsh, Gabriella Shinyoung Song, Hammad A. Khan, Prem Parmar, Kaitlin T. Vance, Gillian Grant, Giulia Mesaroli, Judith Hunter, Kyle Vader

**Affiliations:** a Department of Physical Therapy, University of Toronto, Toronto, Ontario, Canada; b Toronto Academic Pain Medicine Institute, Women’s College Hospital, Toronto, Ontario, Canada; c Department of Rehabilitation Services, The Hospital for Sick Children, Toronto, Ontario, Canada; d School of Rehabilitation Therapy, Queen’s University, Kingston, Ontario, Canada; e Chronic Pain Clinic, Kingston Health Sciences Centre, Kingston, Ontario, Canada

**Keywords:** Chronic pain, physiotherapy, interprofessional, chronic pain clinic, qualitative research

## Abstract

**Background**: Chronic pain affects one in five persons and is a leading contributor to years lived with disability and high health care costs. In 2016, the government of Ontario increased public funding for pediatric and adult hospital-based interprofessional chronic pain clinics (HICPCs) in Ontario, Canada, expanding the role of physiotherapy in chronic pain management in the province. This role has yet to be described in the literature.

**Aim**: The aim of this study was to explore physiotherapy practice within HICPCs in Ontario.

**Methods**: We conducted an interpretive description qualitative study based on semistructured interviews with physiotherapists employed in pediatric and adult HICPCs in Ontario. Interviews were audio recorded, transcribed verbatim, and reviewed for accuracy. We analyzed interview data using thematic analysis.

**Results**: Ten physiotherapists who practiced in pediatric and adult HICPCs (*n* = 4 pediatric; *n* = 6 adult) in Ontario were interviewed between February and April 2020. We constructed five themes related to physiotherapy practice in this setting. Themes included (1) contributing a functional lens to care; (2) empowering through pain education; (3) facilitating participation in physical activity and exercise; (4) supporting engagement in self-management strategies; and (5) implementing a collaborative approach to whole-person care.

**Conclusions**: Our results illuminate how physiotherapy practice within HICPCs in Ontario focuses on providing a collaborative and whole-person approach to care, with an emphasis on supporting patients to increase their functional capacity by promoting engagement in active chronic pain management strategies.

## RÉSUMÉ

**Contexte**: La douleur chronique touche une personne sur cinq et est l’un des principaux contributeurs au nombre d’années vécues avec un handicap, ainsi qu’aux coûts de soins de santé élevés. En 2016, le gouvernement de l’Ontario a augmenté le financement public des cliniques interprofessionnelles de la douleur chronique (HICPC) pédiatriques et pour adultes en milieu hospitalier en Ontario, Canada, élargissant le rôle de la physiothérapie dans la prise en charge de la douleur chronique dans la province. Ce rôle n’a pas encore été décrit dans la littérature.

**Objectif**: Le but de cette étude est d’examiner la pratique de la physiothérapie au sein des HICPC en Ontario.

**Méthodes**: Nous avons mené une étude qualitative descriptive interprétative fondée sur des entrevues avec des physiothérapeutes employés dans des HICPC pédiatriques et pour adultes en Ontario. Les entrevues ont fait l’objet d’un enregistrement audio, puis elles ont été transcrites textuellement et vérifiées pour assurer leur exactitude. Nous avons analysé les données des entrevues en ayant recours à une analyse thématique.

**Résultats**: Dix physiothérapeutes qui ont exercé dans des HICPC pédiatriques et pour adultes (n = 4 pédiatriques; n = 6 pour adultes) en Ontario ont été interviewés entre février et avril 2020. Nous avons construit cinq thèmes liés à la pratique de la physiothérapie dans ce contexte. Les thèmes comprenaient (1) l’apport de l’angle fonctionnel aux soins; (2) l’autonomisation grâce à l’éducation sur la douleur; (3) faciliter la participation à une activité physique et à l’exercice; (4) soutenir l’engagement dans des stratégies d’autogestion; et (5) la mise en œuvre d’une approche collaborative des soins à la personne globale.

**Conclusions**: Nos résultats illustrent la façon dont la pratique de la physiothérapie au sein des HICPC en Ontario se concentre sur une approche de soins collaborative et globale, qui met l’accent sur le soutien aux patients pour augmenter leur capacité fonctionnelle en favorisant l’engagement dans les stratégies actives de prise en charge de la douleur chronique.

## Introduction

Chronic pain is defined as pain persisting or recurring for greater than three months in duration^1^ and is common in both pediatric and adult populations, with an estimated prevalence of one in five.^2,3^ Chronic pain conditions are among the leading contributors to years lived with disability worldwide.^4^ The financial costs associated with chronic pain, including direct health care expenditures and costs due to lost work productivity, are estimated at 56 to 60 billion dollars per year in Canada.^5^

Historically, persons with chronic pain were treated by a single health care discipline (e.g., a primary care physician) in settings that lacked interprofessional collaboration.^6^ However, the effectiveness of this approach has been questioned by both health care providers and persons accessing these services.^7–9^ As a result, primary care physicians often make referrals to specialized chronic pain clinics that involve an interprofessional team of health care professionals with expertise in chronic pain management.^10^

There is high-quality evidence supporting the efficacy and cost-effectiveness of interprofessional chronic pain management in comparison to unimodal management with regards to pain intensity, return to work outcomes, and subsequent use of health system resources.^11,12^ Given the efficacy and cost-effectiveness of interprofessional chronic pain care, in addition to a desire to combat the opioid crisis, public funding for pediatric and adult chronic pain clinics was increased in Ontario, Canada, in 2016.^13^ This increase in funding resulted in the expansion of previously established chronic pain clinics and the creation of new clinics across the province. In Ontario, most publicly funded chronic pain clinics are hospital based and consist of an interprofessional team of health care professionals, including physicians, nurses, psychologists, social workers, occupational therapists, and physiotherapists.^14^ In order to access these services in Ontario, persons with chronic pain require a referral from their primary care provider (e.g., a family physician or nurse practitioner) or a specialist physician.^14^ Because these services are publicly funded, persons with chronic pain are able to access these programs without any direct out-of-pocket costs.^14^

Though physiotherapy approaches to managing chronic pain and pain-related disability have been described in the literature, including graded physical activity,^15^ self-management support,^16^ and psychologically informed practice,^17^ there is a gap in understanding how these approaches are being applied within hospital-based interprofessional chronic pain clinics (HICPCs) in Ontario.

Peng and colleagues have previously described the most frequent services provided by physiotherapists within multidisciplinary pain treatment facilities in Canada, including assessment, individualized physiotherapy and exercise, and transcutaneous electrical nerve stimulation.^18^ However, this research was conducted prior to the expansion of the physiotherapy role within HICPCs in Ontario and was based on questionnaire data and, as such, did not include in-depth qualitative perspectives on physiotherapy practice in this setting. To our knowledge, no published research has qualitatively explored physiotherapy practice within HICPCs in Ontario.^13^

The purpose of this study was to explore physiotherapy practice within HICPCs in Ontario.

## Methods

### Study Design

We conducted an interpretive description qualitative study.^19^ Interpretive description seeks to “provide a thematic or integrative description of a phenomenon of applied or practical interest.”^19(p38)^ We selected an interpretive description methodology for this research because this approach is well suited for qualitative research that has practical implications in the context of health care.^19^ Ethics approval for this study was obtained from the Health Sciences Research Ethics Board at the University of Toronto in Toronto, Canada (Protocol No. 38448).

### Research Team

The research team included six master of science in physical therapy students (LT, RMW, GSS, HAK, PP, KTV) who had received training on qualitative research, three physiotherapists employed within pediatric and adult HICPCs in Ontario (GG, GM, KV), and one faculty advisor at the University of Toronto in the Department of Physical Therapy who has a program of research focused on chronic pain (JH).

### Participants

Physiotherapists were eligible to participate in this research study if they were (1) currently employed as a physiotherapist in a pediatric or adult HICPC in Ontario and (2) able to complete an interview in English either in person or remotely over the telephone or through videoconference.

### Recruitment

We used a convenience sampling technique to recruit physiotherapists from pediatric and adult HICPCs in Ontario.^20^ A recruitment e-mail was sent to a senior health consultant at Ontario Health, who then forwarded the recruitment e-mail to physiotherapists employed in HICPCs across the province of Ontario. Interested physiotherapists then contacted the student researchers by e-mail, at which time they were provided with additional study details, including the letter of information and consent form for the study. Eligible participants were then invited to schedule an interview via telephone, videoconference, or in person based on participant preference.

### Data Collection

We developed a semistructured interview to understand physiotherapists’ perspectives toward their practice within HICPCs in Ontario (see Box 1).^21^ The semistructured interview guide was piloted with a member of the research team who was actively employed as a physiotherapist within an adult HICPC in Ontario. Based on the pilot interview, minor revisions were made to the semistructured interview guide to improve clarity, enhance flow, and decrease redundancy. Each participant also completed a demographic questionnaire. Remote interviews (telephone and videoconference) were conducted by three student researchers (LT, RMW, GSS). Nine participants completed telephone interviews, and one completed a videoconference interview. At the beginning of the interview, informed consent was obtained verbally from each participant before conducting the demographic questionnaire and the semistructured interview. The interviews were audio recorded and transcribed verbatim by a student researcher. All transcripts were subsequently reviewed for accuracy by another member of the student research team.

**Box 1.** Questions from semistructured interview guide

(1) How would you describe your role as a physiotherapist within an HICPC?
(2) Can you walk me through a typical day working as a physiotherapist in an HICPC?
(3) What does a typical client/patient look like that you provide care to as a physiotherapist in an HICPC?
(4) How is the physiotherapist role in an HICPC different from the physiotherapist role in other practice settings (if any)?
(5) How is the physiotherapist role in an HICPC similar to the physiotherapist role in other practice settings (if any)?
(6) Describe for me what your assessments look like as a physiotherapist in an HICPC.
(7) Describe for me what your treatment/follow-up sessions look like as a physiotherapist in an HICPC.
(8) Describe for me any nonclinical duties you engage in (if any) as a physiotherapist in an HICPC (i.e., research, education, or managerial).
(9) What are the rewarding parts of your work and what are the challenging parts of your work as a physiotherapist in an HICPC?

HICPC = hospital-based interprofessional chronic pain clinic.

### Analysis

**Figure 1.**
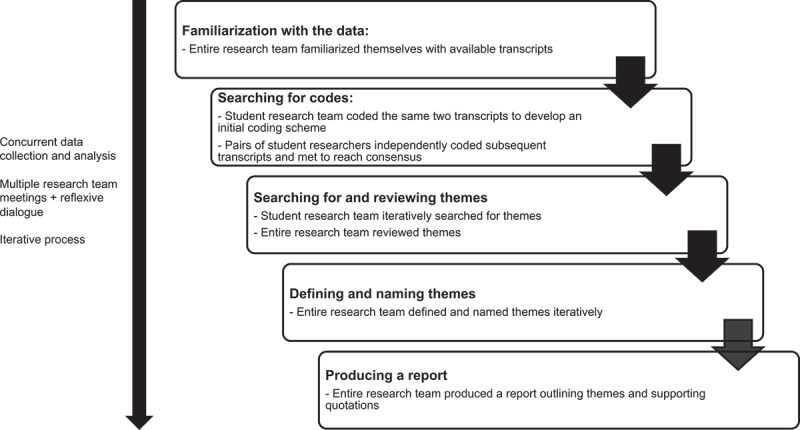
Visual display of analysis process

Data collection and analysis occurred concurrently. Our analysis was guided by Braun and Clarke’s approach to thematic analysis, which outlines six steps: (1) familiarization with the data; (2) generating initial codes; (3) searching for themes; (4) reviewing themes; (5) defining and naming themes; and (6) producing a report.^22^ As a first step, the entire research team (LT, RMW, GSS, HAK, PP, KTV, GG, GM, JH, KV) reviewed the available transcripts to familiarize themselves with the data. Next, the student researchers (LT, RMW, GSS, HAK, PP, KTV) initially coded the same two transcripts individually, representing one pediatric and one adult HICPC setting. Using these two transcripts, the student researchers then came to consensus to develop a codebook with descriptions of each code, to facilitate a common approach to coding across the research team. After the first two transcripts were coded, the entire research team met to discuss the coding scheme and engage in reflexive dialogue. The student researchers subsequently divided into partner groups, with each transcript being coded individually first and then by consensus between the two assigned student researchers using NVivo 12 (QSR International)^23^, continually adding and refining codes through an iterative process. Each transcript was independently coded by two student researchers to ensure comprehensiveness of the coding process. Throughout the analysis process, the entire research team met frequently to discuss potential themes, which were reviewed, defined, and named through an iterative process until the final report was produced. See Figure 1 for a visual display of our analysis process.

### Rigor

We implemented multiple strategies to enhance rigor^24^ and establish trustworthiness of the results using the following processes: (1) piloting the interview guide with a physiotherapist employed within an HICPC to ensure applicability to the participant population; (2) sending deidentified interview transcripts to each individual participant to ensure accuracy of the transcript via member checking; (3) coding transcripts individually and subsequently reaching consensus on codes between pairs of student researchers; and (4) engaging in ongoing collaboration and reflexive communication between the student research team and clinical/faculty advisors via frequent team meetings throughout data collection and data analysis processes. It is important to note that although we sent interview transcripts to each participant as a form of member checking, we did not provide participants with the study results to provide feedback upon.

## Results

A total of ten physiotherapists who practiced within pediatric and adult HICPCs in Ontario (*n* = 4 pediatric; *n* = 6 adult) were interviewed between February and April 2020. Interviews lasted between 45 and 75 min. See Table 1 for details on participant demographic information.

**Table 1.** Participant demographic characteristics (*N* = 10)

Characteristic	*n* (%)
Sex Female Male	9 (90) 1 (10)
Employment status Permanent Contract	10 (100) 0 (0)
Full-time equivalents 0.2 0.4 0.6 0.8 1.0	0 (0) 0 (0) 2 (20) 2 (20) 6 (60)
Years of practice as a physiotherapist 0–5 years 6–10 years 11–15 years 16–20 years >20 years	1 (10) 3 (30) 2 (20) 1 (10) 3 (30)
Years of practice in an HICPC 0–5 years 6–10 years 11–15 years 16–20 years	9 (00) 0 (10) 0 (0) 1 (10)
Country of entry-level physiotherapy education Canada International	10 (100) 0 (0)
Use of controlled acts None Acupuncture (including dry needling) Spinal manipulation Internal pelvic floor physiotherapy	6 (60) 2 (20) 1 (10) 1 (10)
Population demographic (adult vs. pediatric) Adult Pediatric	6 (60) 4 (40)

HICPC = hospital-based interprofessional chronic pain clinic.

**Figure 2.**
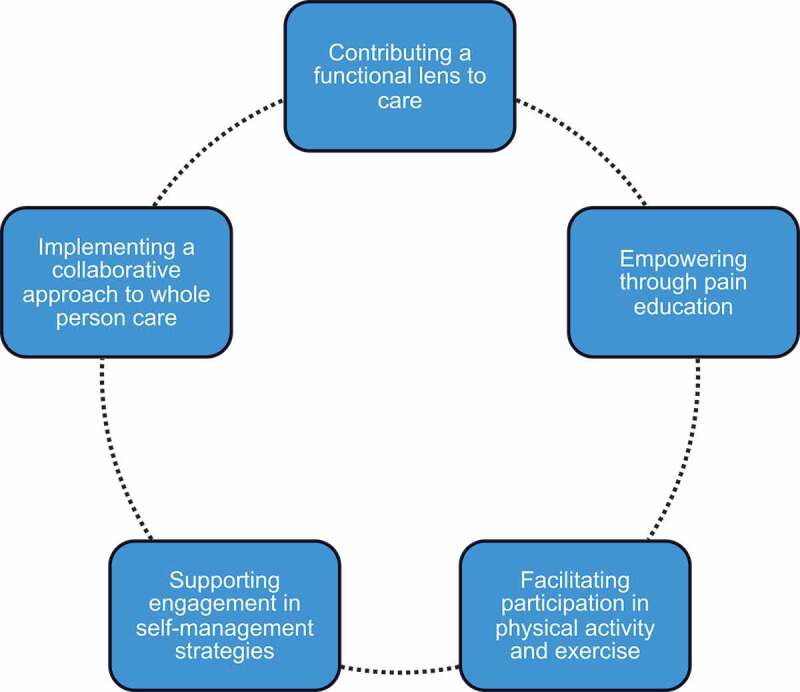
Visual representation of themes related to physiotherapy practice within HICPCs in Ontario

We constructed five themes related to physiotherapy practice within HICPCs in Ontario: (1) contributing a functional lens to care; (2) empowering through pain education; (3) facilitating participation in physical activity and exercise; (4) supporting engagement in self-management strategies; and (5) implementing a collaborative approach to whole-person care. See Figure 2 for a visual representation of these themes. Additional supporting quotations, beyond those included in this article, are provided in Supplemental File 1.

### Contributing a Functional Lens to Care

This theme describes how physiotherapists contribute a functional lens to care within HICPCs. Within their day-to-day practice, participants described how they focus on function in both the assessment and treatment of their patients. Participants reported that instead of emphasizing pain reduction, their ultimate goal is to improve functional outcomes and quality of life among their patients by supporting meaningful engagement in activity and participation in daily life.

Participants shared how they assess their patients through a functional lens, with an emphasis on how chronic pain has impacted activity and participation:

So from a physio point of view we really look to assess how the pain has impacted [patients] physically. So their ability to do sort of their [activities of daily living], basic mobility like ambulation, transfers, up to their participation in gym class or other age-appropriate physical activities, recreational physical activities. (P10)

Participants also described that they strive to establish patient-reported goals for treatment that focus on engagement in meaningful activities:

[We do] a lot of goal setting, so again that involves [setting] more functional goals, returning to meaningful activities for the patient, so rather than focusing on pain reduction or elimination we want to focus on […] something like being able to go for a walk with the dog, being able to tolerate standing for whatever minutes to wash the dishes. (P08)

By promoting meaningful engagement in activities, participants described how they hope to improve quality of life among their patients:

[As physiotherapists …] we’re all trying to work with patients to get [them to do activities that] ultimately gives them quality of life. (P09)

Ultimately, this functional lens was described by participants as a uniting element in physiotherapy practice within the context of interprofessional chronic pain care:

I think as […] physios [in chronic pain care] our mandate is to get patients […] to become more functional once again. (P05)

### Empowering through Pain Education

This theme describes how physiotherapists within HICPCs empower their patients through pain education. Participants reported that they regularly provide education on pain to their patients, with a particular focus on the biopsychosocial nature of chronic pain and the fact that pain is not always proportional to the level of tissue damage. Importantly, participants described how they integrate pain education throughout all of their sessions; with the goal of empowering their patients to engage in active chronic pain management strategies.

Within their day-to-day practice, participants reported that they regularly provide pain education:

[We give our patients] a ton of education on what chronic pain is, the difference between hurt and harm. (P03)

Participants described how they use pain education as a strategy to reduce fear of movement and improve confidence among their patients to engage in active chronic pain management strategies, such as physical activity:

I spend a lot of time in education around reassurance of movement and how it’s safe to do these movements and chronic pain is not [proportional to] the level of tissue [damage …] it’s [about] the nervous system. (P08)

Participants also stressed that pain education was not a stand-alone or one-time intervention. Rather, participants reported that pain education is integrated throughout every session they provide with their patients:

There’s always an element of pain neuroscience education; [patients] need to understand about their pain in order to help move treatments along. Someone who’s more informed and who understands what they’re doing and the meaning of pain, of chronic pain, is really important, so in every session education is a thread to everything we do. (P05)

Although pain education was described as an important component of physiotherapy practice within HICPCs, participants also acknowledged that changing beliefs related to chronic pain can be a very challenging endeavor:

There isn’t a great understanding of pain in the general population … one of our big goals [as physiotherapists] can be trying to change someone’s belief system [about chronic pain], and that can be really challenging. (P07)

### Facilitating Participation in Physical Activity and Exercise

This theme describes how a core component of physiotherapy practice within HICPCs involves facilitating participation in physical activity and exercise. When considering the context of chronic pain, participants described how they facilitate participation in physical activity and exercise using a tailored approach, whereby they focus on patient preferences, goal-oriented exercise, graded activity, mind–body approaches, and establishing connections with community-based services.

Participants described how their recommendations for physical activity and exercise are based on patient preferences:

The type of exercise depends on what the [patient] is interested in. So some [patients] might do the pool or they might do yoga or this or that. (P10)

Participants also reported that they make sure that exercise has a clear link to a patient-reported goal:

[We talk about] goals and what [the patient is] hoping to work towards. And then […] we’ll start with a few exercises that are related to whatever [the patient’s] goals are. (P07)

When facilitating participation in physical activity and exercise, participants described the importance of taking a graded approach with their patients, with a focus on time-based pacing and gradually increasing activity tolerance over time:

So our exercise prescription and technique check on how to do them based on […] time-based pacing […] we want to create success with patients so we want to give an appropriate level such that it’s not making the pain worse […] and then we gradually [increase] the parameters. (P08)

Participants also described how they integrate mind–body approaches to physical activity and exercise, such as Qi gong or yoga, with the goal of “rewiring” or “remapping” a patient’s nervous system:

I sort of see [the physiotherapy role as] trying to be a guide or almost like a bit of a coaching position where we’re trying to encourage, um, people to explore movement in a safe way so that we can help them remap their nervous system or rewire their nervous system. (P07)

In addition, participants reported that they strive to connect their patients with community-based services; with the ultimate goal of supporting adherence in physical activity and exercise over the long term:

For some patients, it’s helping connect them to a community resource. We have a municipal program here, so for some patients, I help them get a free gym membership, so they can access a community center [for exercise]. (P04)

### Supporting Engagement in Self-management Strategies

This theme describes how physiotherapists within HICPCs focus on supporting engagement in self-management strategies. As part of their routine practice, participants reported that they support their patients to implement evidence-based chronic pain self-management strategies in their daily lives, including physical, mind–body, and behaviorally based strategies. By supporting engagement in self-management strategies, participants expressed a desire to increase their patients’ sense of control over their chronic pain.

Participants reported that they empower their patients to implement evidence-based self-management strategies that can be used on a daily basis:

[T]here’s […] a focus on self-management and empowering the patient and really prioritizing evidence-based therapies. (P09)

Participants described how they often provide their patients with physically based self-management strategies:

I mean, and some of the exercises I show people how to do their own myofascial release. Like, you know, using a foam roller, a soft foam roller or massage balls, I show them how to do their own muscle release techniques. (P06)

Participants also reported that they teach their patients how to implement mind–body self-management strategies:

We teach a lot of […] belly breathing, muscle relaxation, different sort of […] body scans, breath awareness scans. (P10)

In addition, participants described how they support their patients to engage in behavioral self-management strategies, such as goal setting:

[G]oal setting, activity tracking, and sort of, understanding their own, uhm, their own current, sort of, baseline of activity and ability is part of it. (P09)

Ultimately, participants indicated that by supporting engagement in self-management strategies, their goal is to provide patients with improved control over their chronic pain:

I’m really building a program that they’re able to do independently […] and have them practice that and take ownership and control. (P03)

### Implementing a Collaborative Approach to Whole-Person Care

This theme describes how physiotherapists implement a collaborative approach to whole-person care within HICPCs. Participants reported that they implement a whole-person approach to care by addressing biopsychosocial contributors to chronic pain in collaboration with their interprofessional colleagues. Furthermore, by delivering patient sessions jointly with other interprofessional health care providers and collaborating with their colleagues toward mutually agreed-upon goals of care, participants described how they provide holistic care that considers the whole person.

Participants described how they implement a whole-person approach to care by addressing the biopsychosocial drivers of chronic pain collaboratively with their interprofessional colleagues:

[Our care is] done in a holistic way. Because pain is biopsychosocial, any psychosocial contributors must be addressed simultaneously, and although a lot of that is done by other practitioners, there’s an element of that that overflows into physiotherapy and should be addressed as well. (P02)

When describing how they enact a whole-person approach within their practice, participants often shared how they focus on psychological and cognitive contributors to chronic pain, such as fear of movement:

[As physiotherapists we’re] well situated to help with a mind–body outlook because we can be facilitators of change, because we have skill sets that could be well utilized for, you know, motivational interviewing, [cognitive–behavioural therapy]-like strategies within our scope of practice from a functional rehab[ilitation] perspective. Minimizing fear in order to promote movement. (P05)

Participants also demonstrated a whole-person approach to care by delivering sessions for patients jointly with other interprofessional health care providers:

The psychologist will come to the rehab gym. […] So [the psychologist] would basically in that time be helping [a patient] use a mind–body strategy while they’re doing a challenging physical task [during our physiotherapy visit]. So helping them sort of either calm their body, calm their mind. Doing some cognitive intervention in preparation or during something that’s very challenging physically. (P10)

Finally, participants exemplified a whole-person approach to care by collaborating with their interprofessional colleagues toward shared goals for patient care:

We do rounds every week, so when a client starts […] we will sit down and talk about goals so that maybe sleep is something, but [occupational therapy is] working on positioning and I’m trying to calm down some shoulder pain so it doesn’t wake them up when they roll over and the psychologist is working on PTSD and how to calm down the nervous system to fall back asleep, to keep their mind quiet, but the common goal is sleep, we’re just coming at it from different approaches. (P01)

## Discussion

To our knowledge, this is the first qualitative study to explore physiotherapy practice within HICPCs in Ontario. Our results provide a foundation for understanding physiotherapy practice within this setting and build upon previous research that has broadly described services provided by physiotherapists within interprofessional chronic pain care in Canada.

Peng and colleagues have previously described the most frequent services provided by physiotherapists, along with other interprofessional health care providers, within multidisciplinary pain treatment facilities in Canada.^18^ However, this research was conducted prior to the expansion of the physiotherapy role within HICPCs in Ontario in 2016, and results were limited to questionnaire data. For instance, although Peng and colleagues described how physiotherapists commonly provide assessment, individualized physiotherapy and exercise, and transcutaneous electrical nerve stimulation,^18^ these results lacked specific details of physiotherapy practice within the context of interprofessional chronic pain care. As such, the results of our study build on this previous work by providing in-depth qualitative data related to physiotherapy practice within this setting.

We found that physiotherapists within HICPCs in Ontario bring a functional lens to care, whereby their primary goal is to improve functional outcomes and promote engagement in meaningful activities among their patients. The *International Classification of Functioning, Disability, and Health* by the World Health Organization describes function as “an umbrella term encompassing all body functions, activities and participation.”^25(p3)^ Instead of emphasizing pain reduction, participants described the value of focusing on function, with a particular focus on activities and participation, when establishing patient-reported goals for care. This finding aligns with previous literature that has documented the value and evidence base of interventions that focus on improving function within interprofessional chronic pain management programs.^26^

Participants also reported that pain education is a common thread that is integrated throughout all of their clinical encounters with patients. Moseley and Butler have endorsed the idea that “explaining pain” is an important component of chronic pain management,^27^ which has been supported in the literature.^28^ A recent meta-analysis by Watson and colleagues found that pain education has clinically relevant impacts on kinesiophobia.^29^ This aligns with findings in our study, whereby participants described how they use pain education as a strategy to decrease fear of movement and promote engagement in active chronic pain management strategies, such as physical activity and exercise.

Considering the challenges that adults with chronic pain can face when participating in physical activity and exercise,^30^ our results highlight the important contribution of physiotherapists in this setting by providing individualized support related to physical activity and exercise. Within this study, participants described the importance of taking a tailored approach when supporting engagement in physical activity and exercise, including an emphasis on graded activity and facilitating connections with community-based resources; both of which have been identified as recommendations for health care providers when promoting participation in physical activity and exercise from the perspective of adults with chronic pain.^31^ Similar to previous work by Scott-Dempster and colleagues, participants described the value of using graded activity as a strategy to “rewire” the nervous system.^32^ Furthermore, participants’ emphasis on facilitating patient connections with community resources not only aligns well with a chronic disease management approach^33^ but is specifically supported by Dnes and colleagues’ research highlighting that adults with chronic pain want their health care providers to make connections with community-based exercise programming.^34^

Previous work by Hutting and colleagues has described the role of physiotherapists in promoting the use of self-management strategies for people with chronic musculoskeletal disorders.^35^ Within our study, participants indicated that they strive to support and empower their patients to implement various chronic pain self-management strategies, including physical, mind–body, and behavioral strategies. These types of self-management strategies are consistent with those included in previously established physiotherapist-led chronic pain self-management programs.^16^ Because persons who engage in active self-management strategies for their chronic pain are less likely to have high levels of pain-related disability,^36^ there is value in physiotherapists across settings continuing to support their patients to engage in active chronic pain self-management strategies.

Participants in this research described how they implement a collaborative and whole-person approach to chronic pain care. Our results provide a different perspective than previous research by Cowell and colleagues, who identified that physiotherapists in primary care lack confidence in addressing psychological factors in the management of chronic low back pain.^37^ Within our research, participants expressed how they strive to consider and address psychological factors within their practice. This difference may be because of the collaborative nature of HICPCs in Ontario, whereby physiotherapists are able to learn skills from their interprofessional colleagues, including psychology, social work, and occupational therapy. Physiotherapy care that recognizes and adapts treatment based on psychological factors is often referred to in the literature as psychologically informed care.^17^ Interestingly, although participants described how they consider and address psychosocial drivers of chronic pain, most often this had a psychological or cognitive focus, such as addressing fear of movement. Participants less often described providing socially informed approaches to care that focused on cultural sensitivity and social determinants of health, which has been identified as a critique of the biopsychosocial model.^38^ This finding also aligns with previous research by Toye and colleagues, who conducted a meta-ethnography to understand health care professionals’ experience of treating adults with chronic pain, who describe the challenge of navigating the juxtaposition of biomedical and biopsychosocial models.^39^ Considering the important impact of social factors on pain,^40,41^ physiotherapists may benefit from critically reflecting how they can implement more socially informed approaches to their practice within this context. Considering the interprofessional nature of HICPCs in Ontario, whereby physiotherapists provide care alongside social workers, occupational therapists, and psychologists, there may be opportunities for physiotherapists to learn from their interprofessional colleagues in order to improve their competence in this area of practice.

As a whole, participants emphasized how they focus on supporting their patients to engage in active chronic pain management strategies, including pain education, physical activity and exercise, and self-management strategies. It appears that physiotherapy practice within HICPCs in Ontario aligns well with quality standards for chronic pain care. For example, a recent report published by Health Quality Ontario highlights standards for quality chronic pain care, including statements related to goal setting for pain management and function, supported self-management and education, physical activity, and therapeutic exercise.^42^ The concordance between physiotherapy practice and standards for quality chronic pain care is likely because many HICPCs in Ontario are situated within university-affiliated hospitals. As such, participants in our research might have been more aware of up-to-date evidence in comparison to community physiotherapists.

### Limitations

This research has important limitations that need to be acknowledged. First, the target population of this study was limited to physiotherapists employed within HICPCs in Ontario; thus, the scope of physiotherapists’ perspectives who provide chronic pain care outside of this setting is not captured within this article. Second, although this study included physiotherapists who practice within pediatric and adult HICPCs in Ontario, our objective was not to describe the similarities and differences between pediatric and adult physiotherapy care within these contexts. As such, the nuances of practice within pediatric versus adult HICPCs may not have been fully captured or described. Third, this study focused on understanding the perspectives of physiotherapists related to their role in HICPCs in Ontario and did not include the perspectives of patients or other interprofessional team members. Other stakeholder groups may have provided additional details and perspectives regarding physiotherapy practice within this setting that are not described in this article. As such, it would be beneficial for future research to explore physiotherapy practice within this context from the perspective of persons with chronic pain, other interprofessional health care providers, and administrators. Finally, the majority of participants in this research had less than five years of experience practicing with HICPCs in Ontario at the time this research was conducted. As such, it may be worthwhile for future research to explore how physiotherapy practice has evolved in this practice setting as time passes.

## Conclusions

Our results illuminate how physiotherapists within HICPCs in Ontario focus on providing a collaborative and whole-person approach to care, with an emphasis on supporting patients to increase their functional capacity by promoting engagement in active chronic pain management strategies, such as pain education, physical activity and exercise, and self-management strategies. Results may be transferable to other contexts and help to inform the development of physiotherapy roles for the management of chronic pain within other interprofessional health care settings. Future research should understand barriers and facilitators to accessing (e.g., from the perspective of persons with chronic pain) and delivering (e.g., from the perspective of physiotherapists and other interprofessional health care providers) physiotherapy care with interprofessional chronic pain care with the ultimate goal of improving health service delivery in this context.

## Supplementary Material

Supplemental Material
