# Supplementary material for: Exploring physiotherapy practice within hospital-based interprofessional chronic pain clinics in Ontario
Source: Can J Pain. 2021 Apr 29;5(1):96–106. doi: 10.1080/24740527.2021.1905508 (PMC8210862; doi:10.1080/24740527.2021.1905508)
Supplement: Supplemental Material [file UCJP_A_1905508_SM4005.docx]

**Supplemental File 1**

**Additional Supporting Quotations**

| **Theme** | **Supporting Quotations** |
| --- | --- |
| Contributing a functional lens to care | “[The physiotherapist] and physician assessment are pretty similar in terms of we’re gonna do a lumbar scan or cervical scan and look at reflexes and myotomes and dermatomes and general movement. I will take it to more of a functional movement level and our physician tends to focus more on the sensory changes that might happen in a centralized nervous system.” (P01)  “I’ll be looking at strength of some of the larger joints, range of motion of the larger joints, functional movements, so gait, tandem gait, balance assessments probably along with some single-limb stance, single-limb stance with eyes closed, their functional movements, deep squat, reach overhead, I might get them to jump if they’re able” (P02)  “[W]orking to increase their functional abilities [...] and helping them work towards their functional goals.” (P02)  “[T]he underlying goal is to maximize the patient’s functional potential and to work with their … with the patient-centred and family-focused goals.” (P02)  “[I’m] functionally focused.” (P02)  “[I assess] a lot of functional outcomes, so 6 [minute walk test], I do a Tampa Scale of Kinesiophobia on everybody. If it is lower extremity impairment, I do [...] the Lower Extremity Functional Scale [...] functional strength measures including: step ups in 30 seconds, how many crunches they can do in 30 seconds, pushups, plank, bridging, single leg bridging” (P03)  “I think like the overarching goals that physios have of focusing on improving patient or client function.” (P03)  “I’d say the most rewarding parts are seeing kids return to meaningful activities, that they haven’t done for a while. To being able to, you know, return to attend school full-time when they haven’t in a significant period of time, to pick up uhm meaningful relationships with friends, to return to either sports that they really were interested in before, or explore new activities that they’re getting joy from.” (P03)  “I think as […] physios our mandate is to get our patients [...] to become functional once again […] we’re trying to improve quality of life and functionality.” (P05)  [W]e are so fortunate to have the luxury of time with [our patients] so we know just how much work they need in order to make gains in function and sustain those functional gains.” (P05)  “So for instance my, when I see women for pelvic pain I have a list of questionnaires that is more just for them to describe their function because of their pelvic pain, their pelvic symptoms.” (P06)  “[Our role as a physiotherapist in this setting is] a little bit less specific and targeted than some orthopaedic settings where their goal might be to strengthen a specific area or increase the range of motion in specific area. Umm I think given the patient population that we work with, we’re trying to see them as a whole person rather than focusing uhh very specifically on their areas of pain.” (P07)  “[As physiotherapists we] encourage [our patients] towards function and meaningful activity.” (P07)  “So the rewarding part is when, umm part of what’s quite rewarding about it is that we do get to see people make changes and uhh it’s often people who have been unable to make change for quite some time. And so being able to help people with that is really rewarding and to see those changes happen. And they may not be necessarily changing their pain but um seeing them change how maybe they’re living their lives or what they’re able to participate in.” (P07)  “[W]e focus a lot on like function focus or find meaningful activities they want to return to, so we want to shift the paradigm from like reducing the pain level.” (P08)  “[I try to] get away from like diagnosis focus in [my] mindset and more on like function-focused aspects” (P08)  “[As physiotherapists we] focus on functional tasks, ability to participate in valued activities, and including work, leisure activities, family responsibilities.” (P09)  “[As physiotherapists] we’re trying to, we’re all trying to work with patients to get them, help them do what they, what ultimately gives them quality of life” (P09)  “We do some TENS, we would teach [patients] how to use it, loan them one so that they can check it out and they can see if they like it or not. And a lot of coaching around sort of pairing it with a functional task.” (P10)  “The evidence, so at least for paediatric is towards functional rehabilitation. So not focusing on reducing the pain and pretty much no hands-on therapy at all. So the evidence is towards exercise and function, despite the pain.” (P10) |
| Empowering through pain education | “I always tell the client I’m much more of teacher and a coach.” (P01)  “[We] teach in terms of what is the nervous system, what is its functions, what is pain, what is a nervous system that is wound up and hypersensitive and some of the symptoms [patients] may feel [...] and how we would initiate calming the nervous system.” (P01)  “We explain to [to our patients] that there’s not just one answer to pain, that pain is layered in our emotions, our communication, our eating habits, our postural habits, our sleeping habits, our everyday movement habits, pacing.” (P01)  “[W]e’re essentially delivering, or trying to deliver, a consistent message, you know, through a lot of pain education.” (P04)  “[I take on] more of a coach type role, like a healthcare coach.” (P04)  “[W]e have a pain education session twice a month that’s 2 hours and that’s led by a [physiotherapist] and an [occupational therapist ...] so that’s another form of providing treatment through education.” (P05)  “[T]here’s lots of time spent on the education.” (P05)  “There is [...] a lot of education.” (P06)  “It seems as though on the team I’m the one that’s most comfortable with the explain pain type of education.” (P06)  “[W]e’ll usually do some education, some chatting about pain.” (P07)    “Yeah or like readiness, right? [Some patients are] still really really searching for a diagnosis and there might not be a diagnosis. Often you know their, their motivation in trying to find a diagnosis is cause they may think that a diagnosis means there’s a cure. Right? So if they’re really stuck in that looking for umm an answer that might not exist, they might not be ready to hear umm their sort of pain education type of stuff.” (P07)  “[O]n chronic pain clinics a lot of [the physiotherapy] day-to-day practice involves education, so educating in terms of pain neuroscience for chronic pain, a lot of patient education.” (P08)  “[W]e do a lot of education around pain neuroscience and how um, the level of tissue damage is not proportional to the level of pain you have.” (P08)  “So even doing pain education, we do it through every session. We all have training in motivational interviewing and the language that we use. So, our choice of language, goal setting, all that stuff that’s weaved into your exercise session or your physio session.” (P10)  “We also ask that [...] all [patients] go to a pain education class. They do that in group format and another physiotherapist leads that class. So that is part of her role. And they, it’s a really fun class actually. It’s very interactive, they play games. Lot of like matching games and trivia questions about pain science.” (P10)  “[With pain education] it’s not like it’s one and done. It’s repeated over and over and over again, and discussed over and over again.” (P10) |
| Facilitating participation in physical activity and exercise | “So depending on what the client identifies as the goal, I mean if someone’s got a a a like a radiculopathy I’ll talk them through McKenzie technique, if it’s a frozen shoulder we talk about how to do… identify some of the myofascial stuff that’s going on and stretching and progressing exercise.” (P01)  “We’re trying to return to function and working on individual muscle groups doesn’t necessarily translate to improved function. So, I mean if our starting point has to be strengthening individual muscle groups, well then that’s our starting point, but we’ll move as quickly as we can to greater functional movements. Walking, running, jumping, stairs, playing sports, those sorts of things.”(P02)  “I also see myself as working to increase their functional abilities, increasing their exercise capacity.” (P02)  “[I]f it is a client with any tight muscle, teaching them how to stretch those muscles properly. Doing uhm, lots of exercises focusing on postural control and good posture and improving posture and then integrating function into that good posture as well and progressing that.” (P03)  “So I try to in every session to have some component of cardiovascular exercise, whether it’s on a bike, or MOTOmed, or treadmill, and explaining the rationale for each type. So some component of cardiovascular exercise, and then some strengthening as well as some stretching, if it’s appropriate.” (P03)  “I’ll kind of more focus around the physical activity piece, what they’re doing, what they might be doing kind of activity- or exercise-wise.” (P04)  “[W]e’re the exercise experts. [H]elping people have some more physical literacy [...] and how exercise can be helpful for [...] overall health. Not just for chronic pain and not just for getting stronger, but just for their overall wellbeing.” (P04)  “Most of the treatment involves taking an active approach and guiding [patients] through some sort of physical activity that they can continue on, on their own.” (P04)  “For some patients, it’s helping connect them to a community resource. We have a municipal program here, so for some patients, I help them get a free gym membership, so they can access a community centre.” (P04)  “[W]e’re trying to [...] promote mobility and regular physical activity.” (P05)  “I[‘m] really focused on you know the exercise piece for treatment. And that’s primarily what I do, I would say that’s like 80%, 70% of what I deliver for my interventions.” (P06)  “[I offer groups on] how to exercise with chronic pain and introduction to Qi Gong. Introduction to yoga, chair yoga. And then I [I offer group] exercises to relieve neck and shoulder tension [and to] relieve back and hip tension.” (P06)  “[My goal is] to improve a person’s participation with exercise. To give them a tool to try to help manage their pain or help them feel better [...]. And to, I mean to become a little bit more active with exercise without necessarily aggravating their pain. So doing in that specific way where we do relaxation stuff after we exercise.” (P06)  “[N]ot just your traditional physio exercises five sets of ten. Doing the yoga therapy, doing the Qi Gong.” (P06)  “[M]y one-on-one follow-ups are, it’s usually exercise based. And I would guide them through like, show me what you’re doing or let’s review what you’re doing. I might tweak that exercise, add something new. Whenever possible I send people, I always send people home with a printout of the pictures of exercises.” (P06)  “I really encourage people to think about okay, what at the very end it’s like “What’s my home program?”, and I have them write it down. I’m going to do this exercise three times a week or this exercise once a week, or something like that [...] And I want them to kind of have that experience and to kind of decide that feels good and I encourage them do things that feel good frequently, often to try help with that sensitivity of the nervous system.” (P06)  “physio can kind of be the coach, the coach to steer you in the right direction. And the right direction might be, you know, getting to the point where you’re strong enough to do a community program. I don’t want to see people come to physio for years on end and be dependent on the physio.” (P06)  “There is a lot of exercise prescription and not necessarily traditional exercise prescription. Like getting into things like [...]  yoga and qi qong, because that’s well supported in the research and takes more of that mind-body approach that our patients really need.” (P06)  “And then the follow-up [visits] usually involve thorough reviewing of their exercises. We’re asking about how it went, what were things they faced that was challenging or was helpful about it, umm do they feel like they’re working towards the goal, maybe we wanna revise the goals [...] And progress the exercises. So it might be using that graded approach to make the movement bigger, or to make it more repetitions or more frequent. ” (P07)  “[W]e will make recommendations and we have a list of community resources that we give to patients.” (P07)  “[T]eaching that movements are safe and not dangerous, and prescribing exercises that are both um, doable and appropriate for the patient’s level.” (P08)  “So our exercise prescription and technique check on how to do them based on like time-based pacing [...] we want to create success with patients so we want to give an appropriate level such that it’s not making the pain worse but that it’s success so that will hopefully facilitate more buy-in from the patient, give them more confident that they feel like ‘oh, I can actually do them’ and then we gradually [increase] the parameters.” (P08)  “[As physiotherapists] our unique piece and lens, I think, is generally around mobility and pain management strategies that are, that involve movement in some way.” (P09)  “But for those that maybe come in in more of a readiness for, you know, for action stage or change behaviour, we may focus a bit more on, you know, what are the activities and movements that are meaningful to them and, you know, what’s maybe the, what’s their baseline ability and maybe how can we bridge them from where they are now to where they want to be.” (P09)  “[G]raded activity, which could include daily activities, structured aerobic, strengthening, flexibility, breathing exercises, all those things, and balance exercises actually come into play pretty often.” (P09)  “I think, yeah, there’s always a hope to contribute in some way to a successful transition back to community-based programming [...] so activities related to that could include either looking up or, and sending resources to the patient, sort of at the end of our sessions, or spending time within sessions to sort of look up on uhm, for example [...] community centres’ websites, what’s close to them, what’s affordable and accessible to them. So that’s, that’s something that we spend time doing.” (P09)  “[I]f it’s a 17-year old boy we’re doing something in the gym, they’re lifting weights, doing squats and lunges. We could be doing yoga. We could be doing sort of play based activities with an 8-year old. We’re doing skip rope and ball toss - sort of younger games. We also do a lot of pool therapy, so I could be in the pool. ” (P10)  “80% of the treatment that we do is exercise [...] Pretty much exercise is yeah, the number one thing [...] The type of exercise depends on what the [patient] is interested in. So (like) some [patients] might do the pool or they might do yoga or this or that.” (P10)  “[S]o we might be giving them verbal recommendations on ways to either reduce their fear avoidance, improve their mobility, or increase their independence with mobility or ways to become more physically active, not let pain get in the way.” (P10) |
| Supporting engagement in self-management strategies | “I’m not doing to them a lot of things, if I do a hands-on technique I’m gonna try and figure out how they’re gonna be able to do that for themselves.” (P01)  “I think in our model [...] it’s truly about handing off the self-management strategies.” (P01)  “So I would do assessment and treatment, most of the clients coming through we’re encouraging self management.” (P01)  “I think another big difference is the heavy, heavy focus, from my perspective, on learning self-management [...] with us it’s very much an emphasis on active modalities, very, very little passive modalities used, and we are constantly discussing and talking about transfer of care, either to a local therapist or to self-management and planning things like that from the very first appointment so that they’re aware that this is not meant to be a lifelong relationship, and that they’re meant to take control of their health and wellbeing.” (P02)  “[W]e generally encourage a self-management approach.” (P04)  “I’m pulling out my phone in every treatment session so that we can use downloaded apps for relaxation and breathing but also music.” (P05)  “So I have a bunch of different things that I’ve recorded for patients for their home practice. Chi-gong practice, chair yoga practice, a simple, like, breathing and relaxation practice, like a body scan.” (P06)  “I might teach [patients] how to use certain umm tools that they can learn how to use to self-relie[ve their pain].” (P07)  “And also helps patients identify, that “oh, chronic pain, maybe that does have something to do with the nervous system” because improving, better coping strategies around like stress or changing position frequently did make a difference so it also gives them control over like, “oh I can actually manage my pain flare-up more than I think I could.” (P08)  “So not only the success in terms of improvement but having the ability for the patient to self-manage their pain and then taking your strategies and advice to heart and actually implementing that and having that success […] And then giving them the tools, like the self-management tools that they can have control over.” (P08)  “I would add that self-uh, let’s say self-directed pain management modalities are, you know, can be prioritized, especially for those with, sort of, high levels of pain interfering with their activities. So teaching self-massage, you know, things like heat and cold, and uhm, you know, making recommendations for any [...] what they do as self-management. But self-management is usually the first and foremost.” (P09)  “[G]oal setting, activity tracking, and sort of, understanding their own uhm, their own current, sort of, baseline of activity and ability is part of it, and then” (P09) |
| Implementing a collaborative approach to whole person care | “We regularly as a team attend rounds to communicate and make sure we’re on the same page with goals with clients.” (P01)    “Many clients have a lot of psychological trauma, so communicating at rounds over what keeps the client’s nervous [system] in a safe space, so it might even be this woman can’t sit too close to this man, or the lights have to be on to this level because of this person’s headaches or previous trauma, or the door has to be open. So all those things that might come out in the psychologist’s office then are communicated back to us to use in our own safe approach with the client.” (P01)  “The interpersonal skills and the therapeutic relationships that develop as a result, I think, to be totally honest, is actually where a lot of healing takes place. It’s in that validation, it’s in that trust, it’s in the ability to speak to somebody in a way that conveys that you believe them, that you support what they’re saying, but also to do it in a strict, focused, motivating way to help them want to improve and get better and feel confident that they can and know that they’ve got your confidence that they can and will get better. A lot of those skills I’ve learned from my psychosocial colleagues” (P02)  “I mean also, sorry I should also add, and obviously the interdisciplinary nature of what I do, the fact that I’ve got other clinicians in other disciplines with whom I’m sharing the patient very closely, and have ongoing consistent support, that’s-- that’s not common or typical in the other roles that I’ve worked in, I mean certainly you’d collaborate with other clinicians, but not with the same level of intensity.” (P02)  “[Our care is] done in a holistic way. Because pain is biopsychosocial, any psychosocial contributors must be addressed simultaneously, and although a lot of that is done by other practitioners, there’s an element of that that overflows into physiotherapy and should be addressed as well.” (P02)  “Because pain is biopsychosocial, any psychosocial contributors must be addressed simultaneously, and although a lot of that is done by other practitioners, there’s an element of that that overflows into physiotherapy and should be addressed as well.” (P02)  “Another fantastic part of my job is being able to learn from and collaborate with my colleagues. I’ve learned so much from some of the other disciplines, like social work, psychology, and medicine, that I love expanding my own base of knowledge, I think it only enriches my practice and it enriches what I’m able to do with my patients.” (P02)    “[W]hen a patient reports some sort of impairment with activity, or with a specific skill, the psychologist might be looking at it from a psychological perspective in terms of phobias that may be present or anxiety, and we can be looking at it from a very physical perspective, in terms of perhaps the reason they don’t want to go in the water is because the sensation of the water on the body part’s really upsetting as opposed to a water phobia. Or maybe it’s both things at play, right? But it’s really up to us to kind of explore what about those pieces.” (P02)  “I think the physiotherapist has an important role in being able to destigmatize a lot of the mental health struggles. And introducing some of the non-physical strategies, they don’t have to be, you know, have to go to, you know, a psychologist or a social worker to do a muscle relaxation exercise. Which might be, you know, sort of falls under this mindfulness type of exercise, but I don’t know if that needs to stay in sort of a psychosocial domain.” (P04)  “I think the things that we do well is try and deliver a consistent message where, the physiotherapists can talk about mindfulness and the occupational therapists can encourage safe movement, and it’s more of a unified approach rather than ‘oh you go to the physiotherapist for this, and you go to the occupational therapist for this, and you go to the social worker for this.’ [...] I don’t know that we need to sort of draw the line that physiotherapists can’t do mindfulness or educate patients on pacing and develop strategies for that.” (P04)   “[W]e do some interdisciplinary assessments, where [the physiotherapist] be there for the first visit with the physician or nurse practitioner.” (P04)  “[C]hronic pain is a multifactorial problem and requires a multifactorial solution because of that. So just providing physiotherapy is often not enough, so if patients aren’t, sort of buying in to all the aspects, you know, you’re not going to outrun a, you’re not going to out train or outrun or out lift problems at home or problems with finances or, there’s no exercise or mobilizations for a lot of these problems. Exercise is beneficial, exercise is helpful for mental health, but you’re not going to outrun your borderline personality disorder.” (P04)  “And it’s not always consistent with [who is in an interdisciplinary assessment], you know there might be a social worker, or you know, or plus or minus an occupational therapist. So, for example if there’s an occupational therapist present in the interdisciplinary assessment, I’ll spend way less time asking questions around ADLs and IADLs, they’re going to do a much more through job, they’re a lot better at it than I am. So I’ll kind of more focus around the physical activity piece, what they’re doing, what they might be doing kind of activity- or exercise-wise, uhm, and any time physiotherapy’s involved in the interdisciplinary assessment, we do the physical exam.” (P04)  “I work both within a … interdisciplinary team format and also in an individual basis.” (P04)  “[W]e’re an interdisciplinary team in the most fundamental aspects [...] we’re always reviewing patients from a biopsychosocial perspective, so we’re looking at the entire person and family.” (P05)  “[W]e often do combined treatments [with other interprofessional health care providers] we’re always reviewing patients from a biopsychosocial perspective so we’re looking at the entire person and family.” (P05)    “The psychologist and myself will do joint sessions, mostly to help facilitate the kids being more aware of how to use mind-body strategies in practice, so that we’re both in the gym together, psychologist and myself and then we do joint sessions.” (P05)    “[W]e meet on Friday mornings [as a team] and we have some time set aside for case conferencing, to do case rounds like some difficult case rounds [...] But I can always pick up the phone anytime and call someone being like ‘Okay this person I’m really worried about their mood, you know, like let’s try to, you know how are you seeing them or have you, are you going to follow them or can we get them into the next depression and anxiety group’, or something like that.” (P06)  “[W]e have our case rounds on[ce a] week.” (P07)  “[W]e have a lot of group-based interventions available [that I commonly refer to], and that it really needs team efforts because a lot of the time [patients] coming into our clinic have other psychosocial factors that need to be addressed so I really rely on our team like [occupational therapy], social workers, psychologists to further support the patient.” (P08)  “[A] huge benefit is, is the team aspect and although not completely integrated in all senses, we do work well together where I think we’re a good functioning team uhm, in terms of the uhm, the, you know scheduling of our weekly rounds, which is really crucial to us continuing to work together and communicate about patients.” (P09)  “[P]atients who are referred to our clinic have an interprofessional assessment.” (P10)  “[We use] the three P approach. So like physical, pharmacology and psychology. So physiotherapists tend to represent the physical P of the three P approach. And we kind of see it as like a three-legged stool, where each leg of the stool is just as important as the other and you need all three legs to stand up. So it really is an interprofessional team where everyone is valued I’d say equally.” (P10)    “The psychologist will come to the rehab gym for when there are kids where we’re, we’re really feeling like is gonna be really challenging or physiotherapy, it’s gonna be really hard. The psychologist comes to the gym and we would do the session together. So they would basically in that time be helping them use a mind-body strategy while they’re doing a challenging physical task. So helping them sort of either calm their body, calm their mind. Doing some cognitive intervention in preparation or during something that’s very challenging physically for them.” (P10)  “[W]e’re always talking to the psychologist all day long. Calling, texting, going in their office, we’re constantly communicating. Mostly with the psychologist, also sometimes with the nurses, but a big part of our day is pretty much going back and forth with the psychologist on patient care.” (P10) |
